# Preclinical Application of CEST MRI to Detect Early and Regional Tumor Response to Local Brain Tumor Treatment

**DOI:** 10.3390/pharmaceutics16010101

**Published:** 2024-01-12

**Authors:** Se-Weon Park, Joseph H. C. Lai, Xiongqi Han, Vivian W. M. Leung, Peng Xiao, Jianpan Huang, Kannie W. Y. Chan

**Affiliations:** 1 Department of Biomedical Engineering, City University of Hong Kong, Hong Kong, China; swpark3-c@my.cityu.edu.hk (S.-W.P.); josephlai5-c@my.cityu.edu.hk (J.H.C.L.); xq.han@sibionics.com (X.H.); pengxiao@cuhk.edu.hk (P.X.); 2 Hong Kong Centre for Cerebro-Cardiovascular Health Engineering (COCHE), Hong Kong, China; 3 Department of Diagnostic Radiology, The University of Hong Kong, Hong Kong, China; jphuang@hku.hk; 4 Russell H. Morgan Department of Radiology and Radiological Science, The Johns Hopkins University School of Medicine, Baltimore, MD 21287, USA; 5 Shenzhen Research Institute, City University of Hong Kong, Shenzhen 518057, China

**Keywords:** glioblastoma, CEST MRI, liposome, hydrogel, treatment

## Abstract

Treating glioblastoma and monitoring treatment response non-invasively remain challenging. Here, we developed a robust approach using a drug-loaded liposomal hydrogel that is mechanically compatible with the brain, and, simultaneously, we successfully monitored early tumor response using Chemical Exchange Saturation Transfer (CEST) MRI. This CEST-detectable liposomal hydrogel was optimized based on a sustainable drug release and a soft hydrogel for the brain tumor, which is unfavorable for tumor cell proliferation. After injecting the hydrogel next to the tumor, three distinctive CEST contrasts enabled the monitoring of tumor response and drug release longitudinally at 3T. As a result, a continuous tumor volume decrease was observed in the treatment group along with a significant decrease in CEST contrasts relating to the tumor response at 3.5 ppm (Amide Proton Transfer; APT) and at −3.5 ppm (relayed Nuclear Overhauser Effect; rNOE) when compared to the control group (*p* < 0.05). Interestingly, the molecular change at 3.5 ppm on day 3 (*p* < 0.05) was found to be prior to the significant decrease in tumor volume on day 5. An APT signal also showed a strong correlation with the number of proliferating cells in the tumors. This demonstrated that APT detected a distinctive decrease in mobile proteins and peptides in tumors before the change in tumor morphology. Moreover, the APT signal showed a regional response to the treatment, associated with proliferating and apoptotic cells, which allowed an in-depth evaluation and prediction of the tumor treatment response. This newly developed liposomal hydrogel allows image-guided brain tumor treatment to address clinical needs using CEST MRI.

## 1. Introduction

Glioblastoma multiforme (GBM) is one of the most aggressive primary brain tumors and has a high recurrence [1,2]. Among all gliomas, GBMs account for 54% of all brain tumors, and their five-year relative survival rate is poor, which is less than 5%, especially for the elderly [3]. This is because it is inevitably challenging to remove or kill cancer cells in a continuous and sustainable manner. Local treatments such as Gliadel^®^ [4], which is a round-shaped wafer loaded with chemotherapeutic carmustine and placed in a tumor cavity after surgical resection, have shed light on improving the median survival rate. An increased survival rate of more than 50% in 6 months has been reported. Yet, its limited coverage of the tumor resection site and the resulting edema have compromised the efficacy of the local drug treatment [4,5], which could be owing to the rigidity and degradation of the wafer [6,7]. Considering its downsides, hydrogel could be an alternative carrier that can provide tunable mechanical properties, syringeability, and sustainable drug release [8]. This is because tumor metastasis and migration are highly influenced by biophysical regulators and the environment between the tumor and extracellular matrix [9]. It has been reported that tumor is stiffer than normal cells [10,11,12]. Tumor cells spread and proliferate extensively on a rigid matrix, whereas they fail to migrate on a relatively soft matrix [9,10,13,14,15]. Therefore, biomaterials that are relatively softer than the brain are preferred to minimize the migration of tumor cells to neighboring brain regions.

Chemical Exchange Saturation Transfer (CEST) MRI, a non-invasive molecular imaging approach, is based on proton exchanges between targeted molecules and the water. It detects many exchangeable protons in vivo and has been applied in imaging molecular alterations in brain tumors [16,17], stroke [18], Alzheimer’s disease [19,20], and multiple sclerosis [21]. It is especially unique for brain tumor imaging, which detects natural protons of many endogenous molecules and drugs [16,17,22,23]. Two distinctive CEST contrasts, amide proton transfer (APT) at 3.5 ppm and a relayed nuclear Overhauser effect (rNOE) at −3.5 ppm, are well known for brain tumor diagnosis [17,24,25,26,27,28]. Considering that tumors have high cell density and proliferative cells, both APT and rNOE show differences between the normal brain region and the tumor region. Clinical CEST brain tumor assessment focuses on the MTR_asym_, which considers both APT and rNOE together [28], while some studies focus on these two CEST contrasts independently [26]. An increase in the APT signal is strongly associated with tumor proliferation and grade, while the rNOE contrast originating from aliphatic protons has a negative correlation with tumor grades, which could be attributed to a decrease in phospholipids [21,25,26,29,30]. In addition to endogenous contrasts, exchangeable protons on biomaterials and drugs could be detected by CEST. We and others have demonstrated that chemotherapeutics, liposomes, and hydrogel microbeads are CEST-detectable [8,23,31]. For example, gemcitabine-loaded chitosan-dextran (CD) hydrogel generated CEST contrasts at 1.1 and 2.2 ppm in the mouse brain [31]. These studies demonstrate that multiple CEST contrasts could be specific label-free biomarkers for tumor and biomaterial characterization.

With these considerations, we aim to develop liposomal hydrogel and monitor brain tumor treatment longitudinally using CEST MRI, where controlled drug release and low rigidity can be achieved. Moreover, given that CEST allows us to assess treatment efficacy [32], we hypothesized that molecular change could be detected before the tumor volume change and regional tumor analysis could be achieved without the aid of biopsy. This could greatly help clinicians to understand the tumor treatment status further so that the following diagnosis or treatment can be determined more efficiently. Our approach could be a promising theranostic application for the local brain tumor treatment, which uses multiple CEST contrasts.

## 2. Materials and Methods

### 2.1. Preparation of Drug-Loaded Liposomal Hydrogel

Liposomes were prepared using thin-film hydration method. DPPC, cholesterol, and DSPE-PEG2000 were mixed at a molar ratio of 1.17:0.51:0.02 and 0.49:1.19:0.02 for cholesterol content of 30% and 70%, respectively, and weight concentration of 25 mg/mL. The solution was dried on a rotary evaporator at 30 °C and 30 rpm to form a homogeneous thin film layer. Afterward, 1 mL of gemcitabine solution (20 mg/mL, pH 7.0) was added and sonicated under 55 °C to form a lamellar liposome solution. For the control group, 1 mL of PBS (pH 7.0) was added instead of a drug solution. The liposome solution was filtered through a 400 nm polycarbonate filter using an extruder. For unencapsulated gemcitabine, it went through a gel column containing Sephadex G50 pre-equilibrated with DI water. The final liposome solution was stored at 4 °C.

For hydrogel fabrication, 5 mg/mL of methotrexate and 1 wt% of alginate powder were added to the resulting solution and hydrated overnight at 4 °C. Moreover, 4.32 wt% of calcium D-gluconate solution was mixed with a volume ratio of 1:10. Using three-way stopcock, the mixture was mixed homogeneously and centrifuged to remove bubbles. The hydrogel was stored at 4 °C. The illustrative figure of drug-loaded liposomal hydrogel can be found in Supplementary Figure S1.

### 2.2. Liposome Characterization, Drug Loading Determination

The size and polydispersity index (PDI) of liposomes were measured using dynamic light scattering (DLS) using Zetasizer (Malvern Instruments, Worcs, UK). The particle concentration was measured by Nanosight (Malvern Instruments, Worcs, UK) at room temperature. For drug loading determination, liposome solutions were treated with surfactants Triton Tween-20 (0.05 *v*/*v*%) solution to disrupt the liposome structure and release the inner payload completely. After diluting to proper concentration, UV absorbance at 258 nm was measured. The following concentration was determined using the calibration curve of gemcitabine solutions.

### 2.3. Hydrogel Rheology Studies

All rheological measurements were conducted on a rheometer (NETZSCH, Bayern, Germany) using a parallel-plate configuration with a 20 mm diameter and a gap of 0.5 mm. Dynamic oscillatory frequency sweep tests were performed from 0.1 to 10 Hz at room temperature with a 1% strain amplitude after equilibration.

### 2.4. Drug Release Test

All hydrogels were centrifuged to remove bubbles before drug release test. A quantity of 0.2 mL of hydrogel was added with 1.6 mL of PBS (pH 7.4) on top surface of the hydrogel and placed on shaker at 37 °C and 60 rpm. At each time point, 0.2 mL of supernatant was taken out and replaced with fresh PBS. The supernatant was sonicated at 45 °C for over 20 min, and UV absorbance of gemcitabine and methotrexate was measured at 268 nm and 303 nm, respectively.

### 2.5. Cytotoxicity Test

U-87 MG glioma cells (ATTC, Manassas, VA, USA) were cultured in DMEM supplemented with 10% FBS and 1% penicillin-streptomycin. Cells were cultured in culture flasks (T-25) and incubated at 37 °C and 5% CO_2_. In order to study cytotoxicity, U-87 MG glioma cells were seeded in 96-well (2000 cells/well) plates. The culture medium was replaced after overnight incubation, and 20 μL of hydrogel and PBS were added to each well. A quantity of 20 μL of MTT assay was added and incubated for 2 to 4 h. Using a microplate reader (Molecular Devices, San Jose, CA, USA) at 490 nm, the number of cells was quantified.

### 2.6. MR Imaging

A 3T Bruker Biospec system (Bruker, Ettlingen, Germany) was used for MR imaging. For the phantom imaging for liposomal hydrogels, a 40 mm volume coil was used at 37 °C. For CEST acquisition, the rapid acquisition with refocused echoes (RARE) as a readout module, and the parameters follow as below: saturation power (B_1_) = 0.8 µT with continuous-wave (CW), saturation time (t_sat_) = 3000 ms, repetition time/echo time (TR/TE) = 6000/72.84 ms, slice thickness = 2 mm, image size = 64 × 64, field of view (FOV) = 35 × 35 mm^2^, RARE factor = 32, and saturation frequency varied from −10 to +10 ppm with a total number of saturation frequencies of 86. Three M0 images with saturation frequency offset at 300 ppm were acquired. Therefore, the total scan time was 17 m 12 s.

For in vivo imaging, an 82 mm quadrature volume resonator serving as a transmitter and a single surface coil serving as a receiver were used for mouse brain imaging. Mouse anesthesia was achieved with isoflurane at 1.5–2% for induction and 1% for respiration maintenance at 30 BRPM during the MRI scan. A warming pad at 37 °C was set to maintain its body temperature. For T2-weighted acquisition, the parameters are as below: TR/TE = 2000/97.05 ms, FOV = 20 × 20 mm^2^, image size = 256 × 256, and scan time = 3 min 12 s. For CEST acquisition, the sequence was also a RARE-based CW CEST, and the parameters are as below: B_1_ = 0.8 µT, t_sat_ = 3000 ms, TR/TE = 5000/5.9 ms, slice thickness = 1 mm, image size = 96 × 96, FOV = 18 × 18 mm^2^, and RARE factor = 32. Saturation frequency varied from –15 to +15 ppm with total number of saturation frequencies of 97. Four M0 images with saturation frequency offset at 200 ppm were acquired. Therefore, the total scan time was 24 m 15 s. A low saturation power (0.8 µT) was used to minimize other confounding effects, such as direct water saturation (DS) and magnetization transfer contrast (MTC).

The in vitro and in vivo data were post-processed using custom-written Matlab code. The CEST contrast (%) was calculated by using Lorentzian difference analysis (LDA) [16,33,34], in which Z-spectrum was subtracted from the Lorentzian fitted water spectrum. Three CEST contrasts at 3.5 ppm (APT), 2.4 ppm (amine), and −3.5 ppm (rNOE) were extracted for analysis.

### 2.7. Animal Protocol

All animal experiments were approved by Department of Health (DH) of Hong Kong and complied with the Regulation of Animals (Control of Experiments) Ordinance (Chapter 340, Department of Health, Hong Kong). All animal experiments were performed in Laboratory Animal Research Unit (LARU) of City University of Hong Kong. Food and water were provided in a pathogen-free condition with free access and controlled by LARU.

Seven female and five male NOD-SCID (6–8 weeks) mice were anesthetized using 1–1.5% isoflurane in oxygen at 1–1.5 L/min. U87 MG glioma cells were cultured in DMEM supplemented with 10% FBS and 1% penicillin-streptomycin. Cells were cultured in culture flasks (T-75) and incubated at 37 °C and 5% CO_2_. For cell inoculation, cell with density of 0.5 million/3 μL using Hamilton airtight syringe (10 μL) was injected with a flow rate of 0.3 μL/min with a coordination of 2.0 mm lateral, 0.2 mm anterior from the bregma and 3.8 mm deep. After injection, syringe was maintained for 10 min and slowly withdrawn. After two weeks, when the tumor volume reached around 2 mm^3^, mice were injected with hydrogel with a flow rate of 0.3 μL/min and a coordination of 2.2 mm lateral, 0.2 mm anterior from the bregma, and 2.3 mm deep. The tumor volume (mm^3^) was calculated based on tumor size (mm^2^) × slice thickness (1 mm).

### 2.8. Histological Examination

On day 10, mice were anesthetized and perfused with saline (0.9%) and 10% neutral buffered formalin (NBF) to fix brain tissues. The brain tissues were resected, post-fixed in NBF overnight, transferred to sucrose solution (30 wt%), and kept at 4 °C. Moreover, 14 μm sections were cut on a cryostat (Leica, Wetzler, Germany) and directly mounted onto the microscopic slides.

Hemotoxylin and Eosin (H&E) staining was performed according to the standard protocols. For Ki-67 staining, after rehydration in PBST, antigen retrieval was performed for 20 min. Slides were incubated in BlockAid Blocking Solution (#B10710, Thermofisher, Waltham, MA, USA) for 1 h and followed by primary antibody incubation (1:200, #MA514520, Thermofisher, Waltham, MA, USA) overnight. Secondary Antibody (1:1000, #A32754, Thermofisher, Waltham, MA, USA) was used for 2 h and mounted with DAPI. The manufacturer’s staining protocol was followed for TUNEL staining (AB206386, Abcam). Microscopic and fluorescence images were acquired with bright field and fluorescence microscope, respectively.

## 3. Results

### 3.1. Liposomal Hydrogel with Sustainable Release and Low Rigidity

**Figure 1:**
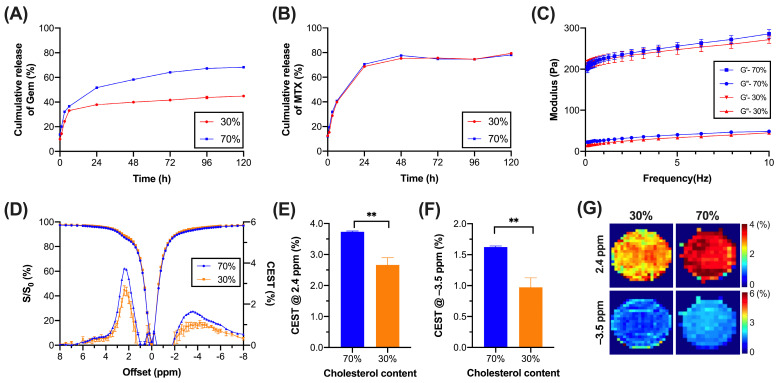
Characteristics and CEST contrasts of liposomal hydrogels with 30% and 70% cholesterol formulations. Cumulative release of (**A**) gemcitabine and (**B**) methotrexate loaded into the liposome and hydrogel, respectively, over 5 days. (**C**) Frequency sweep measurements of liposomal hydrogels (*n* = 3 per group). (**D**) Z-spectrum (left *y*-axis) and corresponding CEST signal (right-*y*-axis). CEST contrasts at (**E**) 2.4 and (**F**) −3.5 ppm and their (**G**) corresponding CEST maps. (*n* = 5 per group, data were presented as mean ± SEM. ** *p* < 0.01, Two-tailed *t*-test).

In order to optimize the liposome stability and sustainable drug release, two liposomes with different percentages of cholesterol were fabricated. DPPC combined with different cholesterol molar ratios of 30 and 70% were prepared based on multiple studies in which cholesterol acts as a stabilizer, increases the encapsulation efficiency (EE), and controls drug release [35,36,37]. Moreover, liposomal hydrogel is a favorable platform for the design of this drug delivery with sustainable release [38,39]. Concentrations for both liposomes were set to comparable levels of 2.6 × 10^16^ liposomes/mL. We first observed that higher cholesterol level liposomes showed smaller particle size and more monodisperse distribution (Supplementary Table S1). The particle size and PDI (polydispersity index) of 30%-cholesterol liposome were 245.6 ± 1.8 nm and 0.32 ± 0.01, respectively, while those of 70%-cholesterol liposome were 210.1 ± 2.2 nm and 0.13 ± 0.01, respectively. Moreover, the 70%-cholesterol liposome achieved a higher EE of 84.2 ± 0.2%, while the 30%-cholesterol liposome showed a lower EE of 60.1 ± 0.1%, which could be attributed to different molar ratios of cholesterol during liposome fabrication. Then, we performed release studies for two CEST-detectable anticancer drugs, gemcitabine, and methotrexate, over 5 days, where those drugs have been demonstrated to have synergistic therapeutic effects on tumors [40]. Since gemcitabine was encapsulated into the liposome and methotrexate was mixed with hydrogels after liposome fabrication, the release profiles for two anticancer drugs showed distinct trends. On day 5, gemcitabine in 30% and 70% cholesterol liposomes was released by 44.9 and 68.3%, respectively (Figure 1A), where liposomes with higher cholesterol molar ratios showed higher cumulative and sustainable drug release. For methotrexate, both liposomes showed comparable release profiles with a burst release on day 1 (Figure 1B).

Regarding rheological properties, two liposomal hydrogels were examined in order to achieve soft hydrogels needed to prevent tumor cell migration and proliferation [9,10,13]. Both liposomal hydrogels showed solid-like hydrogel properties as storage modulus (G′) was higher than loss modulus (G″) (Figure 1C). Although 30%-cholesterol liposomal hydrogel showed a slightly lower modulus than the 70%-cholesterol liposomal hydrogel in both G′ and G″, storage modulus for both hydrogels was in the range of 190–300 Pa, at 0.1 and 10 Hz at 25 °C, which was within the range of storage modulus (0.1 to 600 Pa) of normal brain tissue [11,41,42].

### 3.2. CEST Properties of Liposomal Hydrogel

The CEST contrasts of two liposomal hydrogels were examined using the CEST MRI at 3 T. It is known that amine in gemcitabine and methotrexate generates natural CEST contrasts in the range of 2.0 to 2.4 ppm [23], while aliphatic protons in liposome generate CEST signals at the rNOE range of −3.5 ppm [8,22]. Hence, CEST contrasts at 2.4 and –3.5 ppm were chosen for further study. After obtaining the CEST signal from Z-spectra (Figure 1D), liposomal hydrogels with different cholesterol molar ratios showed distinct differences in CEST contrasts (Figure 1E–G). Specifically, 70%-cholesterol liposomal hydrogel generated higher CEST signals of 3.73 ± 0.03% at 2.4 ppm and 1.62 ± 0.02% at −3.5 ppm than that generated by 30%-cholesterol liposomal hydrogel, i.e., 2.66 ± 0.23 and 0.97 ± 0.15% (*p* = 0.0018 and *p* = 0.0027 respectively). Therefore, a liposomal hydrogel with a cholesterol molar ratio of 70% was selected for the following in vitro and in vivo studies.

### 3.3. Characterization of MGLH and LH

As shown previously, two anticancer drugs and liposomes generated CEST contrasts at 2.4 and −3.5 ppm, respectively [8,23]. CEST MRI was performed to compare the contrast differences between MGLH (methotrexate and gemcitabine-loaded liposomal hydrogel) and LH (free drug-loaded liposomal hydrogel) (Figure S2A–D). The addition of anticancer drugs only led to a difference in CEST contrast at 2.4 ppm, but not at –3.5 ppm, where a high cholesterol liposome formulation enhanced the capacity to accommodate more anticancer drugs [35,36,37], which led to a higher CEST contrast at 2.4 ppm (drugs). At 2.4 ppm, MGLH showed a significantly higher CEST signal than LH (3.73 ± 0.03% versus 0.69 ± 0.03%, *p* < 0.0001). At −3.5 ppm, MGLH and LH showed comparable CEST signals of 1.62 ± 0.02% and 1.77 ± 0.03%, respectively, as the liposome concentrations were comparable in two liposomal hydrogels, i.e., 2.6 × 10^16^ liposomes/mL (Supplementary Table S1). For rheological properties, both hydrogels were soft between 130 and 270 Pa at 0.1 and 10 Hz at 25 °C, but the storage modulus of MGLH was higher than that of LH (Figure S2E). For cell viability, we found that tumor cell viability of MGLH was significantly lower than that of LH (Figure S2F, 32.93 ± 12.52% versus 84.28 ± 15.53%, *p* = 0.0329), indicating a higher anticancer efficacy of MGLH.

### 3.4. CEST MRI of Molecular and Morphological Change in Tumor Region during Treatment

**Figure 2:**
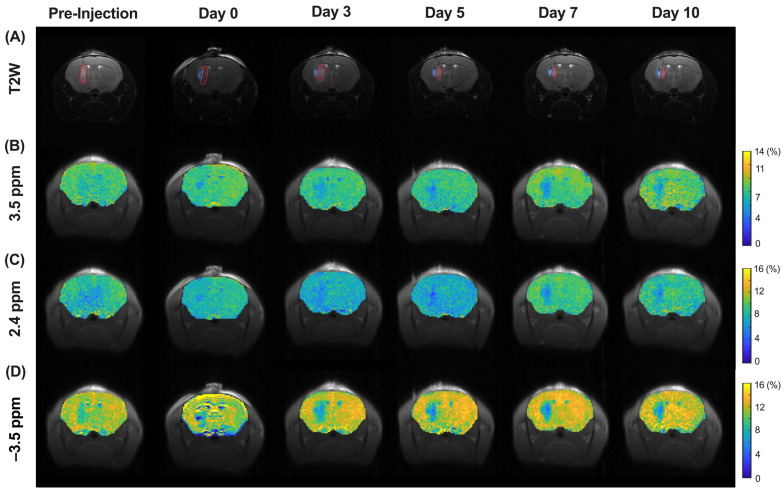
Longitudinal changes of GBM on T2-weighted and CEST maps of the treatment group. From top to bottom: (**A**) T2-weighted images, CEST maps at (**B**) 3.5 ppm, (**C**) 2.4 ppm, (**D**) −3.5 ppm. ROIs in red and blue color indicate tumor and hydrogel, respectively.

**Figure 3:**
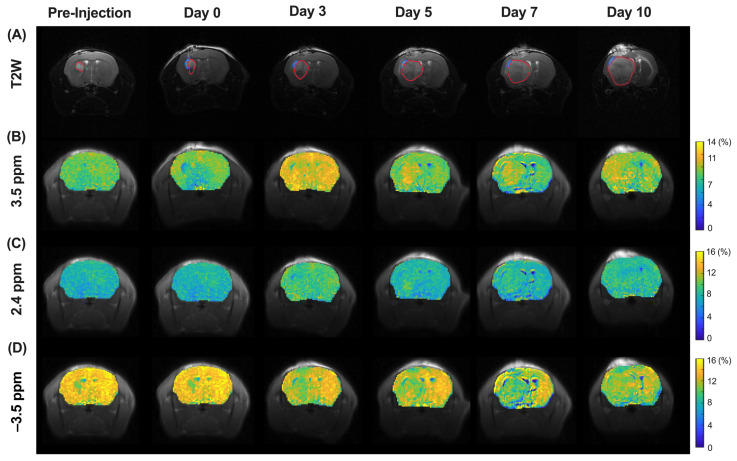
Longitudinal changes of GBM on T2-weighted and CEST maps of the control group. From top to bottom: (**A**) T2-weighted images, CEST maps at (**B**) 3.5 ppm, (**C**) 2.4 ppm, (**D**) −3.5 ppm. ROIs in red and blue color indicate tumor and hydrogel, respectively.

In order to demonstrate the treatment efficacy of liposomal hydrogel, the optimized MGLH was used for in vivo study in the mouse brain. After glioblastoma implantation into the mouse brain, MGLH (treatment group, Figure 2) and LH (control group, Figure 3) were implanted next to the tumor. T2-weighted images (Figure 2A and Figure 3A), together with the corresponding CEST maps (Figure 2B–D and Figure 3B–D), were acquired and presented. The regions of interest (ROI) of the tumor (in red) and hydrogel (in blue) were drawn for further analysis (Figure 2 and Figure 3A), and their CEST contrasts were analyzed separately.

**Figure 4:**
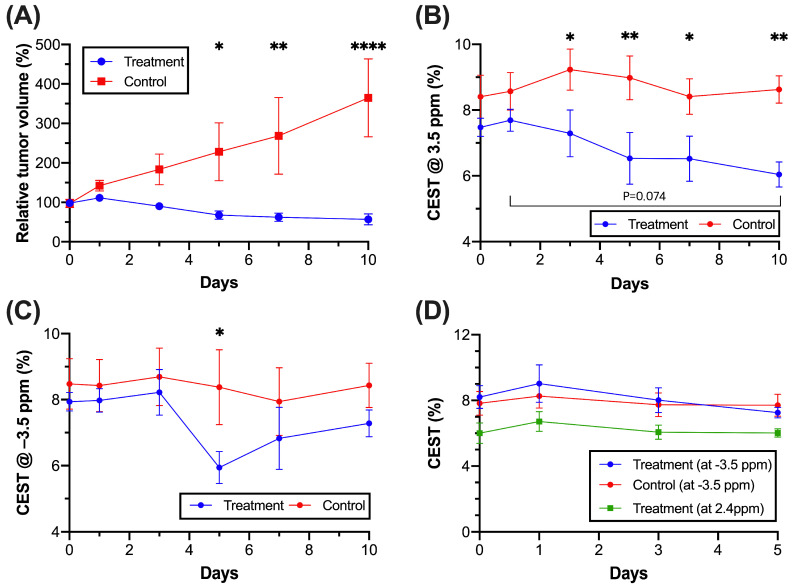
Changes of relative tumor volume and CEST signals between the treatment (*n* = 5) and the control group (*n* = 5–7), with tumor and hydrogel ROIs. (**A**) Relative tumor volume changes at different time points, (**B**) 3.5 ppm, (**C**) −3.5 ppm in tumor ROI, (**D**) −3.5 ppm and 2.4 ppm in hydrogel ROI. (Data were presented as mean ± SEM, * *p* < 0.05 and ** *p* < 0.01, **** *p* < 0.0001, Two-Way ANOVA).

In the T2-weighted image of the treatment group (Figure 2A and Figure 4A), we observed a continuous reduction in tumor volume from day 3, and it was reduced to 56.2 ± 13.8% on day 10, indicating tumor growth suppression. On the contrary, the control group showed a continuous increase from day 1 and dramatically elevated approximately 3.7-fold on day 10. (Figure 4A). A significant increase in the tumor volume was found on day 5 (*p* = 0.0206), day 7 (*p* = 0.0034), and day 10 (*p* < 0.0001). For the CEST contrast of tumors (Table S2), we observed a continuous decrease in the APT signal in the treatment group (Figure 4B), while the treatment group had a significantly lower APT signal than that of the control group from day 3 onwards (*p* < 0.05, Figure 4B). From the CEST maps (Figure 3B,D), the tumor region showed hyperintensity at 3.5 ppm but hypointensity at −3.5 ppm when compared to the contralateral region in the control group. Interestingly, in the tumor region, regardless of time points, the CEST signals of the treatment group were lower than those of the control group at both 3.5 and –3.5 ppm. Significant differences were observed on day 3, 5, 7, and 10 (*p* = 0.0249, 0.0052, 0.0339, and 0.0062, respectively) at 3.5 ppm and day 5 (*p* = 0.0373) at −3.5 ppm (Figure 4B,C). In the treatment group, a continuous signal decrease at 3.5 ppm was observed over 10 days as the tumor volume decreased. Most importantly, we observed a significant difference in APT on day 3 between the two groups of mice prior to the observed significant difference in the tumor volume on day 5 (Figure 4A,B), indicating that APT detected an earlier tumor treatment response. At −3.5 ppm, the signal of the tumor in the treatment group was continuously lower than that in the control group during the monitoring stages, with an abrupt signal drop on day 5.

### 3.5. CEST MRI of Liposome and Drug Release during Treatment

In the liposomal hydrogel region (blue), the drug and liposome release was monitored over 5 days using CEST MRI (Figure 2, Figure 3 and Figure 4D). Of note, the hydrogel showed no endogenous CEST contrast when compared to the other regions in the brain; hence, relative drug release could be monitored during treatment. We used 2.4 ppm to indicate drug content and −3.5 ppm to indicate the liposome content of MGLH. For CEST contrast at –3.5 ppm of the liposomal hydrogel region, the treatment and control group showed comparable decrease trends from 9.03 ± 1.14% to 7.25 ± 0.32% and 8.26 ± 0.74% to 7.71 ± 0.67%, respectively, over 5 days (Figure 4D), yet not significant. Similarly, the CEST contrast at 2.4 ppm for two anticancer drugs continuously decreased from 6.71 ± 0.61% to 6.02 ± 0.26% over 5 days.

### 3.6. Regional Tumor Analysis Assessed by Histology

**Figure 5:**
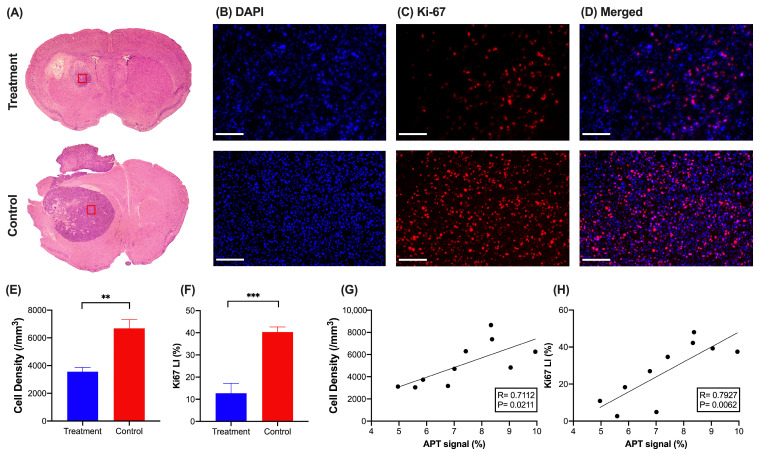
Histology and its evaluation of the GBM in the mouse brain with the treatment and control group on day 10. (**A**) H&E staining of the mouse brain slice for two groups. Region with red box was chosen for cell density calculation. The fluorescence images of (**B**) DAPI, (**C**) Ki-67, (**D**) merged. (Scale bar = 200 µm) (**E**) Cell density and (**F**) Ki-67 labeling index between two groups (*n* = 5 per group). Correlations between APT signal with (**G**) cell density and (**H**) Ki-67 labeling index (*n* = 10). Data were presented as mean ± SEM (** *p* < 0.01, *** *p* < 0.001, Two-tailed *t*-test).

To validate our CEST findings, we stained brain slices with H&E (Figure 5A) and DAPI for assessing cellularity [43], Ki-67 for cell proliferation [32], and TUNEL for apoptotic cells [44] on day 10. The treatment group showed significantly lower cell density than the control group (3555 cells/mm^3^ versus 6683 cells/mm^3^, *p* = 0.0023, Figure 5B,E). Similar to cellularity, proliferating cells of the treatment group (12.75 ± 10.00%) were significantly lower than that of the control group (40.34 ± 5.10%) (*p* = 0.0006, Figure 5C,F). Considering that the APT signal is associated with mobile proteins and peptides [17,27,28], we further studied the correlations of APT contrast with cell density and proliferation. Both cell density and proliferation showed strong correlations with the APT signal (R = 0.7112, *p* = 0.0211 and R = 0.7927, *p* = 0.0062, respectively, Figure 5G,H). Therefore, the APT signal of the tumor in the treatment group was lower than that in the control group, indicating a decrease in cell density and proliferation after the local treatment.

**Figure 6:**
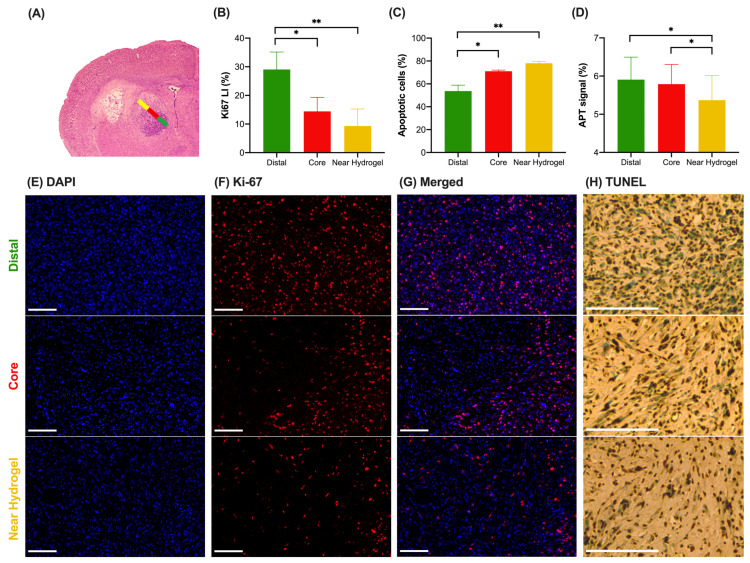
The histopathologic spatial analysis of tumor in the treatment group on day 10 (*n* = 3 per group). (**A**) H&E staining of the mouse brain slice with three regions, indicated by the color bars. Green, red, and yellow regions stand for distant, core, and near hydrogel regions, respectively. (**B**) Ki-67 LI, (**C**) apoptotic cells, (**D**) APT signal of three subregions. The fluorescence images of (**E**) DAPI, (**F**) Ki-67, (**G**) merged, (**H**) TUNEL with three regions (Top: distal region, middle: core region, bottom: near hydrogel region). (Scale bar = 200 µm) Data were presented as mean ± SEM (* *p* < 0.05, ** *p* < 0.01, One-Way ANOVA).

Moreover, we evaluated the treatment efficacy on different regions of the tumor based on the distances between the hydrogel and the tumor using DAPI, Ki-67, and TUNEL staining (Table S3). An exemplary case with three regions, distal, core and near hydrogel regions, marked with different colors is shown in Figure 6A. APT results in three regions defined by the distance from the hydrogel revealed that the furthest tumor region (distal) had the highest APT signal (5.91 ± 0.59%), Figure 6D), which corresponded to the highest cell density (Figure 6E), proliferation (29.06 ± 6.10%, Figure 6F) and the lowest cell apoptosis (53.67 ± 5.21%, Figure 6B,C,H), whereas the region near hydrogel showed the lowest APT signal (5.39 ± 0.65%), cell density and proliferation (9.32 ± 5.94%) with the highest cell apoptosis (78.00 ± 1.53%). Unsurprisingly, the core of the tumor between the distal and near hydrogel zones showed a moderate APT signal (5.79 ± 0.52%), cell proliferation (14.41 ± 4.91%), and cell apoptosis (71.00 ± 1.00%). Contralateral regions showed neither apoptotic nor proliferating cells. (Figure S4). Similar spatial distributions were observed in all animals, and the staining confirmed that the APT signal is highly associated with cell proliferation and apoptosis within different regions in the tumor during treatment.

## 4. Discussion

In this study, we successfully demonstrated a new CEST-detectable liposomal hydrogel, which has superior biochemical and mechanical properties and enables the imaging of the tumor treatment response. This has a great theranostic potential for providing multiple, more independent parameters for clinical evaluations and tumor sizes (Figure 2 and Figure 3). The conventional approach to imaging brain tumors and monitoring their treatment is based on conventional MRI, such as T1 or T2-weighted images with contrast agents, which provides visualization of tumor location and size for further evaluation [45]. Yet, this information does not directly indicate the tumor aggressiveness or tumor response, which makes the prediction of tumor treatment outcomes challenging clinically.

Our CEST MRI findings of the tumor sub-regions supported the fact that the near hydrogel region and the distal hydrogel region showed a distinctive response to the treatment. For example, there were significantly higher proliferating cells (*p* < 0.01) and lower apoptotic cells (*p* < 0.01) in the distal regions than those in the near hydrogel regions (Figure 6B,C). Similarly, APT in the distal region also showed significantly higher signals in distal regions (*p* < 0.05) when compared to the near hydrogel region (Figure 6D). This demonstrated that the molecular alterations detected by CEST MRI were the key to indicating treatment effects. These regional molecular changes could be hard to assess through biopsy, which is only limited to certain regions in heterogenous tumors. Our approach, using image guidance with a change in an APT signal with tumor volume, will provide a more informative evaluation of whether the treatment is effective for all tumor regions.

When we monitored the CEST MRI longitudinally for 10 days, the CEST contrasts of the tumor region at 3.5 ppm enabled us to evaluate tumor response. The APT signal decreases along with tumor volume decrease (Figure 4A,B), which could be attributed to a decrease in endogenous mobile proteins or peptides and a shift in tumor pH caused by anticancer drugs [17,27,28]. The major mechanisms of the action of gemcitabine are abrogating DNA synthesis and self-potentiation by activating deoxycytidine kinase and caspase signaling, leading to apoptosis [46], which has modified tumor microenvironment and ultimately influenced the APT signal. And our histology data shows that our treatment was effective in all tumor regions, as tumor regions’ apoptosis was over 50% (Figure 6C). Moreover, we found that APT could detect earlier tumor responses to the local hydrogel treatment than the tumor volume, indicating that mobile protein or peptide levels exhibited an early response to tumor treatment than the size change, which could be sensitively detected by APT. Strong positive correlations of the APT signal with cell density and proliferation further support that the decrease in APT was sensitive to detecting the reduced tumor cell density and proliferation upon the treatment of anticancer drugs. However, no correlation of the rNOE signal with cell density and proliferation was observed (Figure S3C,D) due to a smaller change in the aliphatic protons of the lipid membrane upon gemcitabine and methotrexate treatment. Both drugs induce cell apoptosis rather than necrosis by inhibiting DNA synthesis [46] and inducing oxidative stress [47]. Nevertheless, the treatment ultimately induced tumor cell death, resulting in a lower rNOE signal in the treatment group than in the control group. Based on the sensitivity of APT toward proliferating cells, CEST can detect early tumor responses toward this local liposomal hydrogel treatment.

Our developed dual drug-loaded liposomal hydrogel has high EE, sustainable drug release, and favorable rheological and mechanical properties to minimize secondary injury and prevent tumor cell migration. For sustainable drug release, we designed two drugs to be released at different rates; gemcitabine, a prodrug, was initially encapsulated inside the liposome and then loaded with methotrexate into the hydrogel for relatively fast release. The two anticancer drugs, gemcitabine and methotrexate, were selected based on treatment efficacy, CEST detectability, and CEST signal intensity. They are widely used anticancer drugs that induce cell apoptosis by inhibiting DNA synthesis and inducing oxidative stress [46,47]. Regarding CEST detectability, drugs, cytidine analogs, gemcitabine, and the antifolate—methotrexate—were considered as they have exchangeable protons of amines and hydroxyl groups and showed good CEST detectability at around 2 ppm [23].

Our study has considered not only biochemical properties but also biophysical properties in that both MGLH and LH were relatively soft compared to the normal brain tissue with storage modulus at a range of 190–300 Pa. This is regarded as the soft-hydrogel range as the storage modulus of normal brain tissue is less than 600 Pa [11,41,42], and this mechanical property is not conducive to tumor cell proliferation and migration [9,10,13,14,15], which opens the possibility to suppress the tumor growth in vivo with the aid of anticancer drugs. This, perhaps, could further improve the treatment outcomes of conventional local brain tumor treatments [4].

CEST MRI showed the possibility of monitoring drug release non-invasively within the hydrogel region for over 5 days at 2.4 and −3.5 ppm. A gradual and comparable decrease of 12% was observed at −3.5 ppm for the release of the liposomes and at 2.4 ppm for the release of both drugs. Given that hydrogel has no background signal with reference to our in vitro release study, there could be ~62% of the drug remaining for sustainable release beyond day 5 at 2.4 ppm, which is supported by continuous tumor suppression up to day 10 in vivo.

A couple of challenges need to be envisaged in this study. First, for monitoring drug release, we were able to obtain a reliable ROI of hydrogel only for the first 5 days of treatment. The hydrogel stayed relatively intact, and it only contained CEST contrasts from the drugs (at 2.4 ppm) and liposomes (at –3.5 ppm) without being interfered by other endogenous CEST contrasts, which are at 1–4 ppm [17,22]. This provides a favorable environment to monitor the dual drug-loaded liposomal hydrogel and its interaction with the tumor. However, we were not able to obtain a reasonable hydrogel region beyond day 5 when the boundary between the hydrogel and tumor became less obvious in the T2-weighted images (Figure 2), i.e., on days 7 and 10. Thus, drug release can only be monitored for a short period of time after treatment. Another limitation is that the tumor volume increased significantly, and the shape of the hydrogel (LH) was distorted by the tumor on days 7 and 10 (Figure 3A) in the control group, so mice had to be sacrificed on day 10. Nevertheless, the CEST contrasts of tumor regions at 3.5 ppm and −3.5 ppm could indicate the treatment efficacy, which enables the monitoring of drug release and tumor response with independent and multiple readouts.

To conclude, we developed CEST-detectable dual drug-loaded liposomal hydrogel for local brain tumor treatment, which had unique drug encapsulating, rheological, and mechanical properties. Importantly, we were able to use multiple CEST contrasts to detect treatment response and release in the tumor and hydrogel regions, respectively. This facilitates an early detection of tumor response and regional tumor response using a distinctive CEST contrast. Our theranostic hydrogel showed a sustainable treatment effect on GBMs over 10 days. The treatment efficacy can be non-invasively assessed. The APT signal detects tumor response 2 days prior to the decrease in tumor volume, which is well correlated with cell density and cell proliferation. Furthermore, different release profiles of drugs and liposomes were detected by the CEST contrasts at 2.4 and −3.5 ppm, respectively, over 5 days. This promising and robust theranostic approach for local brain tumor treatment enables the monitoring of molecular alterations in vivo, which could provide valuable information for enhancing the treatment efficacy in a dynamic and non-invasive manner.

## Data Availability

Data presented in this study are available on request from the corresponding author.

## Supplementary Materials

The following supporting information can be downloaded at: https://www.mdpi.com/article/10.3390/pharmaceutics16010101/s1, Figure S1: Illustrative figure of drug loaded liposomal hydrogel; Figure S2: Characteristics and CEST contrasts of MGLH and LH (*n* = 5 per group); Figure S3: Correlations of CEST signals; Figure S4. The histopathologic analysis of the contralateral region in the treatment group; Table S1: Characteristics of the liposome formulation with different molar ratio of cholesterol (*n* = 3 per group). Size of liposome, polydispersity index (PDI) and encapsulation efficiency (EE%); Table S2: Mean ± SEM and P value of CEST signals of tumor in the treatment (*n* = 5) and control group (*n* = 5–7). (Two-Way ANOVA); Table S3: CEST signals, Ki-67 positive and TUNEL positive within three tumor subregions (*n* = 3 per group). (One-Way ANOVA).

## Abbreviations

CEST	Chemical Exchange Saturation Transfer
MRI	Magnetic Resonance Imaging
APT	Amide Proton Transfer
rNOE	relayed Nuclear Overhauser Effect
MGLH	Methotrexate Gemcitabine encapsulated Liposomal Hydrogel
LH	Liposomal Hydrogel
