# Supplementary material for: Preclinical Application of CEST MRI to Detect Early and Regional Tumor Response to Local Brain Tumor Treatment
Source: Pharmaceutics. 2024 Jan 12;16(1):101. doi: 10.3390/pharmaceutics16010101 (PMC10820766; doi:10.3390/pharmaceutics16010101)
Supplement: Supplementary file 1 [file pharmaceutics-16-00101-s001.zip › pharmaceutics-2795081-supplementary.pdf]

SUPPLEMENTARY DATA

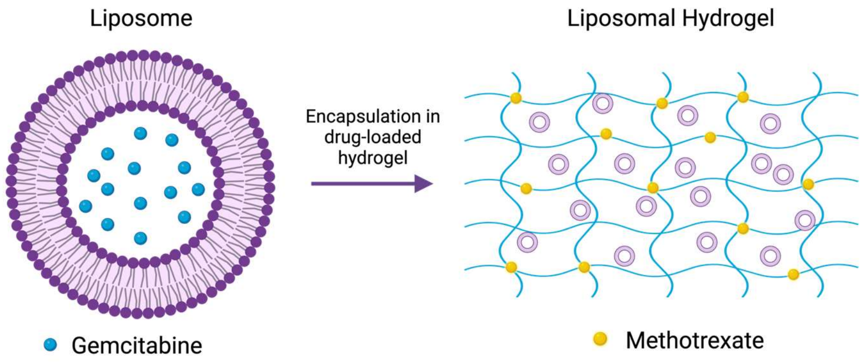

Supplementary Figure S1. Illustrative figure of drug loaded liposomal hydrogel.

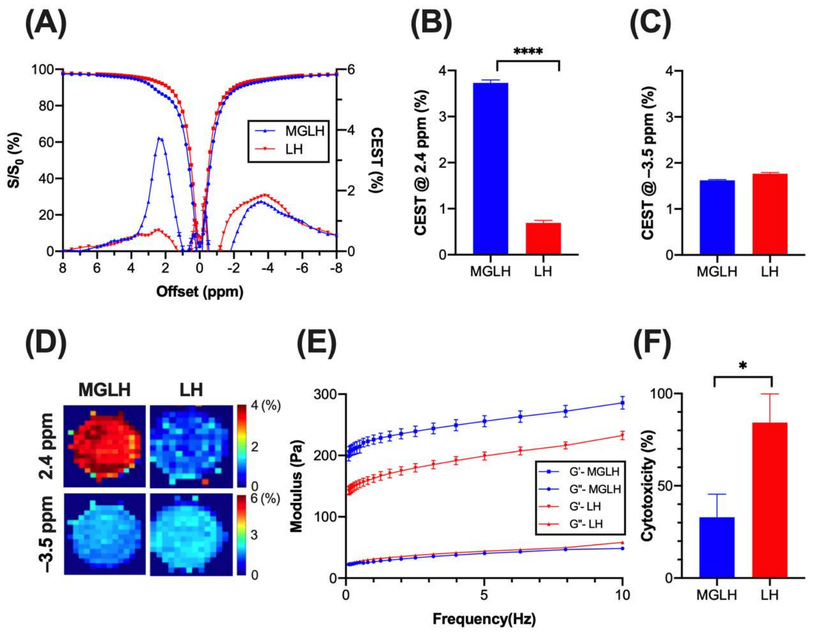

Supplementary Figure S2. Characteristics and CEST contrasts of MGLH and LH (n=5 per group). (A) Z-spectrum (left y-axis) and corresponding CEST signal (right y-axis). CEST contrast at (B) 2.4 ppm and at (C) –3.5 ppm and (D) corresponding CEST maps. (Data were presented as mean ± SEM. ****P<0.0001, Two-tailed t-test) (E) Frequency sweep measurements of liposomal hydrogels. (F) Cytotoxicity test between MGLH and LH (n=5 per group). (*P<0.05, Two-tailed t-tests)

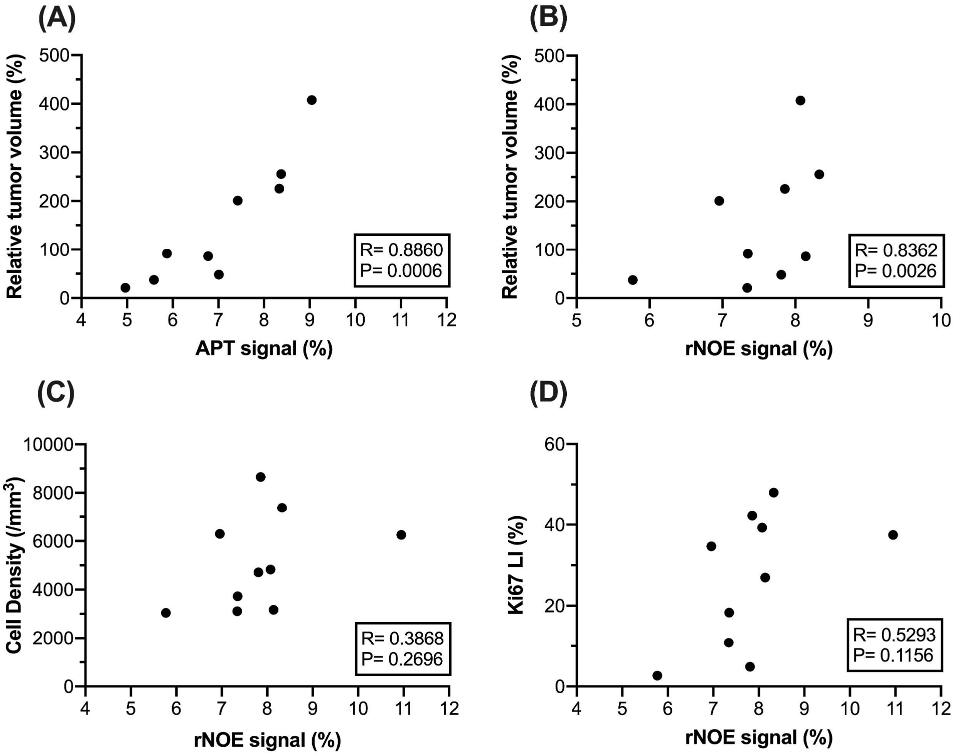

Supplementary Figure S3. Correlations of CEST signals. (A) Correlation between APT signal with relative tumor volume. Correlation between rNOE signal with (B) relative tumor volume, (C) cell density and (D) Ki-67. (n=10). Cell proliferation (%) was calculated based on number of Ki-67 positive cells/ number of nucleus × 100 (%).

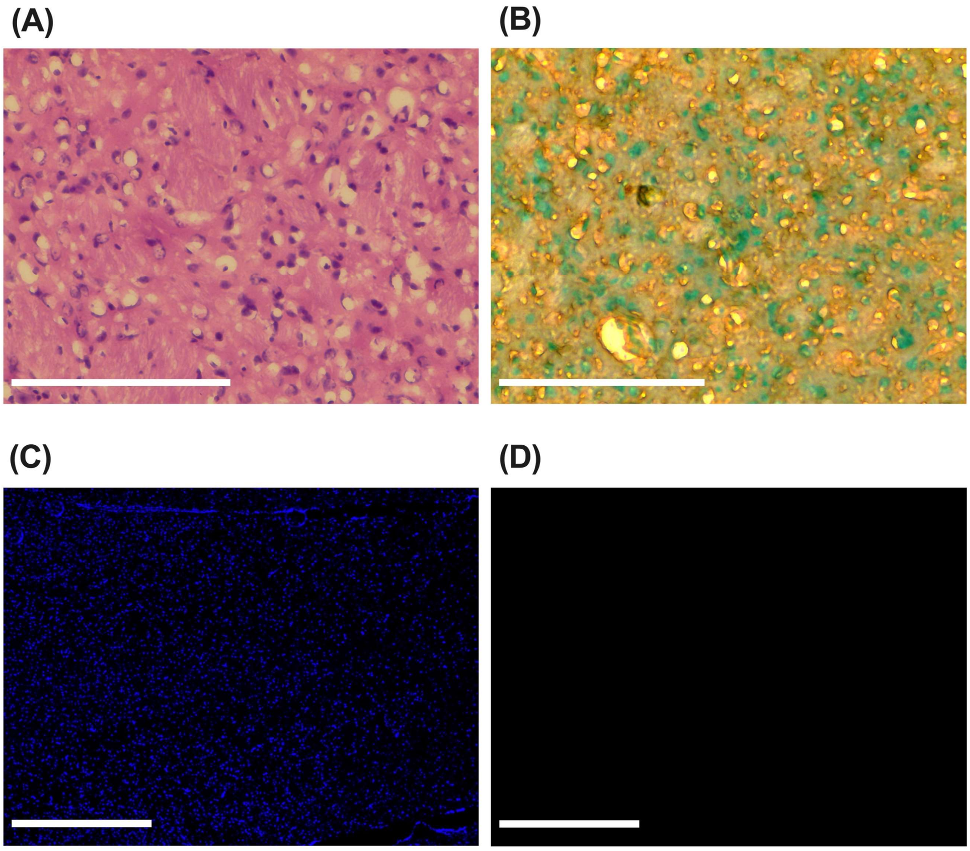

Supplementary Figure S4. The histopathologic analysis of the contralateral region in the treatment group. (A) H&E, (B) TUNEL, (C) DAPI and (D) Ki-67 staining. (Scale bar = 200 μm).

Liposome formulation (DPPC: Chol: PEG)	Size of Liposome (nm)	PDI	EE (%)	Concentration (particles/ ml)
Chol-30 (68.7: 30: 1.3)	245.6 ± 1.8	0.32 ± 0.01	60.1 ± 0.1	$2.64 \pm 0.40 \times 10^{16}$
Chol-70 (28.8: 70: 1.3)	210.1 ± 2.2	0.13 ± 0.01	84.2 ± 0.2	$2.63 \pm 0.42 \times 10^{16}$

Supplementary Table S1. Characteristics of the liposome formulation with different molar ratio of cholesterol (n=3 per group). Size of liposome, polydispersity index (PDI) and encapsulation efficiency (EE%)

Time-points	APT (%)			rNOE (%)		
	Treatment	P value	Control	Treatment	P value	Control
Day 1	7.69 ± 0.34	0.3010	8.57 ± 0.57	7.98 ± 0.36	0.6929	8.43 ± 0.79
Day 3	7.29 ± 0.71	0.0249	9.23 ± 0.63	8.22 ± 0.69	0.6827	8.69 ± 0.87
Day 5	6.53 ± 0.79	0.0052	8.98 ± 0.67	5.94 ± 0.48	0.0373	8.38 ± 1.14
Day 7	6.52 ± 0.68	0.0339	8.41 ± 0.54	6.83 ± 0.94	0.3500	7.94 ± 1.03
Day 10	6.04 ± 0.38	0.0062	8.63 ± 0.42	7.28 ± 0.41	0.3552	8.43 ± 0.67

Supplementary Table S2. Mean ± SEM and P value of CEST signals of tumor in the treatment (n=5) and control group (n=5-7). (Two-Way ANOVA)

	APT (%)	rNOE (%)	Ki-67 (%)	TUNEL (%)	Non-proliferating & non-apoptotic cells (%)
Distal	5.91 ± 0.59	7.14 ± 0.36	29.06 ± 6.10	53.67 ± 5.21	17.28 ± 8.98
Core	5.79 ± 0.52	6.96 ± 0.34	14.41 ± 4.91	71.00 ± 1.00	14.59 ± 4.01
Near Hydrogel	5.39 ± 0.65	7.14 ± 0.63	9.32 ± 5.94	78.00 ± 1.53	12.68 ± 5.53

Supplementary Table S3. CEST signals, Ki-67 positive and TUNEL positive within three tumor subregions (n=3 per group). (One-Way ANOVA)
